# *HNF1B* Transcription Factor: Key Regulator in Renal Physiology and Pathogenesis

**DOI:** 10.3390/ijms251910609

**Published:** 2024-10-02

**Authors:** Eloísa Sánchez-Cazorla, Noa Carrera, Miguel Ángel García-González

**Affiliations:** 1 Group of Genetics and Developmental Biology of Renal Disease, Laboratory of Nephrology, No. 11, Health Research Institute of Santiago de Compostela (IDIS), Clinical University Hospital (CHUS), 15706 Santiago de Compostela, Spain; eloisasancaz@gmail.com; 2 Genomic Medicine Group, Clinical University Hospital (CHUS), 15706 Santiago de Compostela, Spain; 3 RICORS 2040 (Kidney Disease), ISCIII, 15706 Santiago de Compostela, Spain

**Keywords:** *HNF1B*, renal physiology, 17q12 region, gene expression regulation, nephrogenesis, apico-basal polarity, cilia development, cyst formation, renal ion transport, intrarenal metabolism

## Abstract

The *HNF1B* gene, located on chromosome 17q12, encodes a transcription factor essential for the development of several organs. It regulates the expression of multiple genes in renal, pancreatic, hepatic, neurological, and genitourinary tissues during prenatal and postnatal development, influencing processes such as nephrogenesis, cellular polarity, tight junction formation, cilia development, ion transport in the renal tubule, and renal metabolism. Mutations that alter the function of Hnf1b deregulate those processes, leading to various pathologies characterized by both renal and extrarenal manifestations. The main renal diseases that develop are polycystic kidney disease, hypoplastic or dysplastic kidneys, structural abnormalities, Congenital Anomalies of the Kidney and Urinary Tract (CAKUT), and electrolyte imbalances such as hyperuricemia and hypomagnesemia. Extrarenal manifestations include Maturity-Onset Diabetes of the Young (MODY), hypertransaminasemia, genital and urinary tract malformations, Autism Spectrum Disorder (ASD), and other neurodevelopmental disorders. Patients with *HNF1B* alterations typically carry either punctual mutations or a monoallelic microdeletion in the 17q12 region. Future research on the molecular mechanisms and genotype–phenotype correlations in *HNF1B*-related conditions will enhance our understanding, leading to improved clinical management, genetic counseling, monitoring, and patient care.

## 1. Introduction

The hepatocyte nuclear factor 1-beta (*HNF1B*) gene encodes a member of the superfamily of homeodomain-containing transcription factors [1]. These factors interact with promoter regions, coactivators, and co-repressors to regulate gene expression [2,3,4]. *HNF1B* plays a crucial role in the development and function of kidneys, intestines, liver, and pancreatic beta cells [1,3,5,6,7]. In renal processes, *HNF1B* is essential during both embryogenesis (regulating nephrogenesis, cell polarity and cell–cell adhesion) and in the mature kidney (where it regulates electrolyte transport, primary cilia development, and renal metabolism) [3,8,9].

Located on chromosome 17q12, the *HNF1B* gene lies between the 36,046,434 and 36,105,096 positions (GRCh37/hg19) [10]. The 17q12 region is flanked by repetitive sequences, which promotes errors during DNA recombination. If not corrected, these errors result in structural variants such as rearrangements, deletions, or duplications [11,12,13]. The most common alteration in this region is the recurrent 17q12 microdeletion, found in 50% of patients with *HNF1B* mutations. The deletion size varies between 1.3 and 1.8 Mb among patients. In addition to *HNF1B*, this region includes several other genes also affected by the deletion [2,3,14,15], including *AATF*, *ACACA*, *C17orf78*, *DDX52*, *DHRS11*, *DUSP14*, *GGNBP2*, *HNF1B*, *LHX1*, *MRM1*, *MYO19*, *PIGW*, *SYNRG*, *TADA2A,* and *ZNHIT3* [2,3,14,16]. In 30–50% of patients with *HNF1B* mutations, these are *de novo* [14,17].

**Figure 1:**
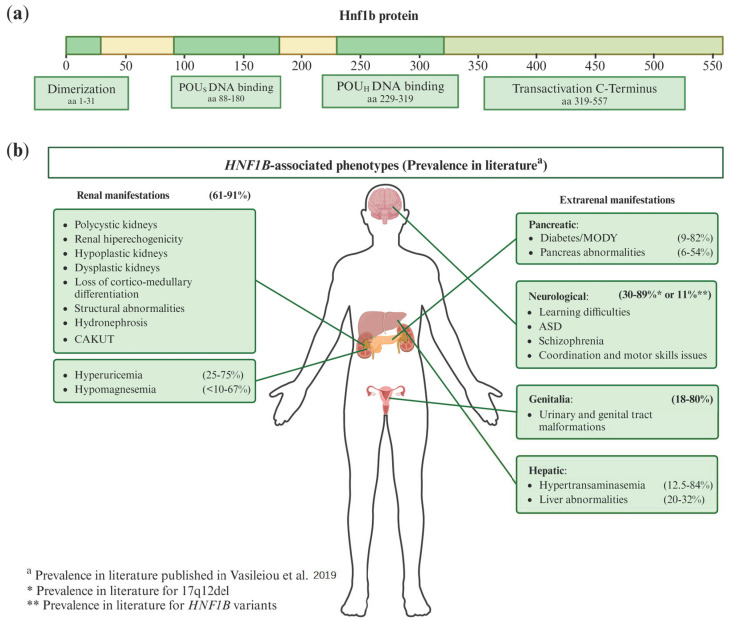
(**a**) Scheme of canonical HNF1B transcript (NM_000458.4) protein domains. aa: amino acid; POU_S_: Pit-Onc-Unc specific; POU_H_: Pit-Onc-Unc homeodomain. (**b**) Phenotypic manifestations associated with *HNF1B* and their prevalence in the literature. There is a substantial variability in both renal and extrarenal manifestations among *HNF1B* patients. These patients may exhibit various combinations of these phenotypes. The prevalence of each manifestation shown in the figure has been derived from the article by Vasileiou et al. [20], where they reviewed the prevalence ranges for each condition from 82 published studies on *HNF1B* patients with clinical data. CAKUT: Congenital Anomalies of the Kidney and Urinary Tract; MODY: Maturity-Onset Diabetes of the Young; ASD: Autism Spectrum Disorder.

The protein encoded by *HNF1B* consists of 557 amino acids, featuring four domains: the dimerization domain, the Pit-Oct-Unc (POU)-specific (POU_S_) DNA-binding domain, the POU-homeodomain (POU_H_) DNA-binding domain, and the transactivation C-terminus domain. The latter is crucial for coactivator recruitment and transcriptional regulation [10,18,19] (Figure 1a).

This gene presents complex animal models, as monoallelic deletion does not produce a clear phenotype in mouse models, whereas biallelic deletion results in a much more severe phenotype than that observed in humans, even causing early embryonic lethality. Niborski L et al. [21] generated a murine model with a human mutation at the intron-2 splice donor site, which exhibits a phenotype more similar to that in humans. Here, the genes previously linked to *Hnf1b* (*Wnt9b*, *Pax2*, *Pkd2*, *Bicc1*, *Crb3*, *Kif12*, *Cys1*, *Glis2*, and *Glis3*) were either modestly decreased or unaffected. Additionally, they observed that HNF1B-regulated gene expression varied by developmental stage; for example, *Umod*, *Tmem27*, and *Pkhd1* were significantly downregulated at E17.5 but not at P1 [21]. This reflects the complexity of the animal models for this gene and the need for ongoing research to better understand *HNF1B* function and its correlation with its phenotype in humans.

This review summarizes the multifaceted roles of the *HNF1B* gene in renal physiology, providing an overview of the current knowledge and highlighting the gene’s significance in kidney development and function.

## 2. *HNF1B*-Associated Diseases and Clinical Significance

*HNF1B*-associated disease follows an autosomal dominant inheritance [22,23]. While biallelic germ line inactivation of *HNF1B* is embryonically lethal [24,25], monoallelic pathogenic mutations cause a variety of disorders in various organs. These disorders often manifest as diabetes and renal conditions during prenatal, postnatal, and adulthood stages [14].

The first described *HNF1B*-associated renal disorder was Renal Cysts And Diabetes syndrome (RCAD), characterized by renal cysts and Maturity-Onset Diabetes of the Young type 5 (MODY5), with high phenotypic variability [2,3]. Renal ultrasound scans of affected patients typically reveal combinations of abnormalities, including polycystic, hyperechogenic, hypoplastic and dysplastic kidneys, loss of cortico-medullary differentiation, structural abnormalities, and hydronephrosis [14,20,22,26,27].

In addition to RCAD, other common renal disorders associated with *HNF1B* include Congenital Anomalies of the Kidney and Urinary Tract (CAKUT) [2,3] and Autosomal Dominant Tubulointerstitial Kidney Disease type *HNF1B* (ADTKD-HNF1B) [28] These are frequently associated with electrolyte disturbances such as hyperuricemia and hypomagnesemia [14,15,19,20,22]. Hypomagnesemia can occasionally lead to a misdiagnosis of Gitelman syndrome [29]. A large proportion of patients reach at least chronic kidney disease (CKD) stage 3 in adulthood, with some developing renal failure [19].

Beyond the kidneys, *HNF1B* mutations can lead to extrarenal manifestations affecting the pancreas (notably MODY [14,15]), liver (hypertransaminasemia, liver cysts), genitalia (genital tract malformations), and neurodevelopment (learning difficulties, autism spectrum disorder, schizophrenia, coordination and motor skills issues or neurodevelopment delay) [15,20,26,30].

Patients may present with various combinations of these phenotypes, as shown in Figure 1b. As illustrated, renal manifestations are the most prevalent, with 61% to 91% of patients displaying at least one renal symptom listed in the figure. The data about prevalence in the literature were derived from the Vasileiou et al. paper [20], where they reviewed 82 published articles containing clinical information on *HNF1B* patients. Differences in study methodologies and the significant phenotypic variability caused by *HNF1B* contribute to the wide range of prevalence rates reported [20].

The phenotypic variability in *HNF1B*-related diseases may be partially explained by the range of biological processes and genes regulated by *HNF1B* at both prenatal and postnatal stages. Section 4 of this review explores these mechanisms and their role in disease development. 

In summary, patients with *HNF1B* mutations display considerable phenotypic diversity, ranging from CAKUT to electrolyte imbalances and extrarenal conditions. The specific symptomatology remains unpredictable, and it is unclear what drives these differences. One possibility is that the type of genetic alteration influences the affected biological pathways, given the regulatory role of *HNF1B* over numerous genes involved in diverse biological processes. Thus, different mutations may disrupt distinct pathways, resulting in diverse clinical outcomes. Recent studies have correlated the type of genetic alteration (17q12 deletion or punctual *HNF1B* variants) with patient symptoms. It has been suggested that patients with punctual *HNF1B* mutations tend to have more severe renal phenotypes and some additional extrarenal manifestations, whereas those with the 17q12 deletion exhibit a broader range of extrarenal symptoms and lighter renal phenotype [19,20,26,31].

Diagnosing *HNF1B*-associated diseases is challenging due to its broad phenotypic manifestations and significant intrafamilial variability in renal phenotypes [14,17]. Further research into the mechanisms underlying this phenotypic variability could improve diagnostic strategies and patient management.

## 3. Hnf1b Protein Domains and Transcriptional Complexes

The structure of the Hnf1b protein consists of four domains (Figure 1a): the dimerization domain (residues 1–31), the Pit-Oct-Unc (POU)-specific DNA-binding domain (POU_S_) (residues 88–180), the POU-homeodomain (POU_H_) DNA-binding domain (residues 229–319), and the C-terminal transactivation domain (residues 319–557). The most genetically conserved regions of *HNF1B* are the dimerization and POU-DNA-binding domains [18,32]. POU_S_ has at least two α-helices. POU_H_ recognizes the consensus sequence 5′-GTTAATNATTAAC-3′ [2,3,4], and forms three α-helices with the third helix functioning as the DNA recognition helix [33]. The POU_S_ and POU_H_ domains cooperate to enhance the DNA-binding affinity and specificity [32,33]. The transactivation C-terminus domain is important for the protein to function [32,33,34]; Tholen L. et al. [34] showed that a Hnf1b lacking this domain failed to induce gene expression due to its inability to bind essential cofactors [34].

To regulate gene expression, Hnf1b forms DNA-binding transcriptional complexes by interacting with other molecules. In these complexes, Hnf1b can function as a homodimer (two Hnf1b molecules) or as a heterodimer with its paralogue Hnf1a [1,23,24], with which it shares 70% sequence homology [35]. Notably, alterations in *HNF1A* are also associated with diabetes [36,37,38,39]. Depending on the target gene, other molecules may join the complex [40]. Hnf1b’s genetic regulation also depends on the tissue and developmental stage, this can be observed in the transcriptomic analysis of *Hnf1b*-altered mice of Niborski L et al. [21] during prenatal (E14.5, E15.5 y E17.5) and postnatal (P1) stages. Their findings revealed differences in the genetic regulation between stages [21].

DNA-binding transcriptional complexes are formed by different combinations of key molecules such as Pcbd1, Pcbd2, CBP, P/CAF, Zyxin, NCoR, and Hdac1 [3,40,41,42,43,44,45].

One of these key molecules is Pcbd1, which interacts with Hnf1b in organs such as the kidney, pancreas, and liver during embryogenesis [40,43,44]; an example of a gene regulated by a transcriptional complex that includes Pcbd1 is *FXYD2* [40,44].

Another key molecule is Pcbd2, which is mainly expressed in the kidney, lung, spleen, and adipose tissue and regulates genes different from those influenced by Pcbd1, such as *KCNJ16* [44].

The key molecules CREB-binding protein (CBP) and P300/CBP-Associated Factor (P/CAF) are coactivators with intrinsic histone acetyltransferase activity, and they are required for Hnf1a- and Hnf1b-mediated transcription [42].

Choi et al. [41] identified zyxin as another key molecule that interacts with Hnf1b in renal epithelial cells [41,45]. It potentially functions as a scaffolding protein that facilitates the assembly of transcription factors and coactivators on gene promoters [41]. Zyxin can interact together with the coactivator CBP, enhancing Hnf1b’s transcriptional activity [41].

The key molecules Nuclear Receptor Corepressor (NCoR) and histone deacetylase 1 (Hdac1) can negatively regulate Hnf1a [42].

Due to the diverse molecular combinations forming its DNA-binding transcriptional complexes, Hnf1b has a broad regulatory scope, controlling the expression of numerous genes with various functions [21]. The composition of these complexes varies depending on the expressed genes, organ, cell type, and developmental stage [21,44,46].

Key molecules forming these complexes could be a significant area of research, potentially leading to the identification of cofactor genes critical for diagnosing *HNF1B*-related pathologies.

## 4. Hnf1b Renal Functions in Kidney Development and Physiology

**Figure 2:**
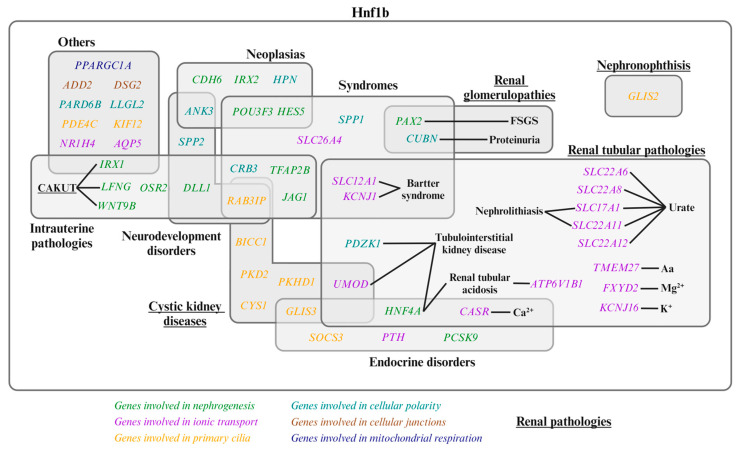
Genes regulated by Hnf1b and their associated diseases. Genes involved in nephrogenesis, ion transport, primary cilia, cellular polarity, cellular junctions, and mitochondrial respiration are shown in green, purple, orange, light blue, brown, and dark blue, respectively. Underlined pathologies are renal pathologies [1,2,3,23,34,45,47,48,49]. CAKUT: Congenital Anomalies of the Kidney and Urinary Tract; FSGS: Focal Segmental Glomerulosclerosis; Aa: amino acid.

As previously mentioned, Hnf1b is a transcription factor that plays a pivotal role in various aspects of kidney development and physiology. Figure 2 illustrates the genes directly regulated by Hnf1b. The mechanistic action of Hnf1b in renal physiology is described in the following four subsections, listed in Table 1.

### 4.1. Hnf1b in Nephrogenesis

**Figure 3:**
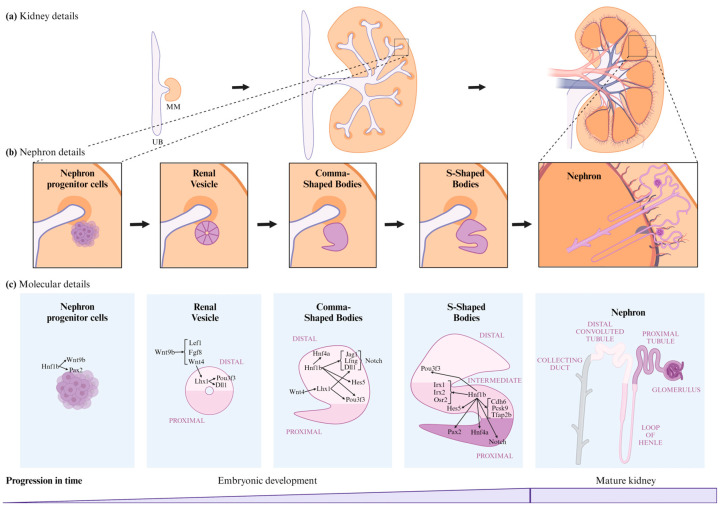
Nephrogenesis stages and Hnf1b’s role in this process. (**a**) Kidney details of renal development from embryonic to mature kidneys. (**b**) Nephron details of renal development from embryonic to mature nephrons. (**c**) Molecular details and Hnf1b’s role in renal development from embryonic to mature nephrons. UB, Ureteric Bud; MM, Metanephric Mesenchyme.

Nephrogenesis is the process by which nephrons develop during embryonic stages until their maturation, at which point they become functional. This process begins with the secretion of signaling molecules from the metanephric mesenchyme (MM) to the ureteric bud (UB), triggering UB outgrowth and the formation of the collecting duct system and ureter (Figure 3a) [2,47,48,50]. The steps of this process, illustrated in Figure 3, involve interactions between transcription factors, promoter regions, coactivators, and co-repressors to regulate gene expression [1,2,3,4]: (1) Induction of nephron progenitor cells to form the renal vesicle (RV) [47,48,50]; (2) morphogenesis and patterning of the RV, which differentiates into the comma-shaped body (CSB) and then the S-shaped body (SSB) [47,48,50]; and (3) final differentiation of the SSB into a mature nephron, which includes the proximal segment (subdivided into Bowman’s capsule, podocyte, and proximal tubule); the intermediate tubule (containing the loop of Henle); and the distal tubule (Figure 3b,c) [48].

1. Induction of nephron progenitor cells to form the RV: Hnf1b is implicated in several stages of renal development, regulating a significant proportion of the mechanisms underlying these processes (Figure 3c). *Hnf1b* conditional inactivation in murine nephron progenitors showed that Hnf1b acts upstream of *Wnt9b* in mice [3,48]. However, research by Niborski L et al. [21] showed minimal impact on *Wnt9b* expression in a mouse model with an identified *HNF1B* human mutation at the intron-2 splice donor site [21]. Wnt9b plays a crucial role in nephrogenesis, particularly in inducing the mesenchyme-to-epithelium transition (MET). Wnt9b is expressed uniformly in the UB epithelium, with increased expression in areas where RVs will form [47,51]. MET is responsible for forming the RV from nephron progenitor cells [2,9,47,51,52,53,54] and involves a stepwise assembly of intercellular junctions and *de novo* establishment of apical–basal polarity to form the RV, the first polarized epithelial precursor of the nephron [55]. This suggests that Hnf1b plays a crucial role in initiating nephrogenesis [3,48].
  Wnt9b signaling activates the expression of the differentiation markers Lef1, Fgf8, and Wnt4 in the surrounding MM [47]. Lef1 and Wnt4 display polarized expression patterns in the distal RV, regulating its early polarity [47]; Wnt4 also triggers the expression of Lhx1, the next transcriptional regulator [47,52,56,57]. Lhx1 drives RV progression to the CSB by activating Dll1 and Pou3f3 (also known as Brn1) expression in the RV. Lhx1 is also involved in the proximo-distal differentiation of the RV, CSB, and SSB [47,48,58]. Hnf1b also regulates *PAX2,* a transcription factor critical for the MET of nephron progenitors, maintaining nephric duct epithelial polarity and SSB differentiation [3,8,47,48,59,60,61,62]. *PAX2* defects have been linked to focal segmental glomerulosclerosis (FSGS) [23].
2. Differentiation into CSB and its progression to SSB: in the CSB, Pou3f3 expression is regulated by Lhx1 in the distal RV and by Hnf1b in the distal CSB regions, as well as the proximal and bulge regions of the SSB [8,47,52]. Pou3f3 is involved in elongating and differentiating the loop of Henle and forming the distal convoluted tubule (DCT) [8,48,52]. Hnf1b also activates Notch pathway components such as Dll1, Jag1, Lfng [52], and Hes5 [8], which are crucial for inducing differentiation and polarization in nephrogenesis [8,47]. Dll1 and Jag1 are ligands for Notch receptors, Dll1 expression is regulated by both Lhx1 and Hnf1b [8,47,48,52], and Jag1 is regulated by Hnf1a and Hnf1b [8,52]. Lfng, regulated by Hnf1b, enhances its expression in the distal region of the CSB and in the proximal region of the SSB [47,48,52]. Defective expression in Notch components significantly reduces proximal tubule formation [8]. In addition, Hes5 expression was observed to change in murine *Hnf1b* mutants [8], and it is specially expressed in the CSB and epithelial cells that form the bulge between mid and lower limb of SSBs [8,48] (Figure 3c).
3. Progression from SSB to mature nephron: Hnf1b regulates the expression of *IRX1*, *IRX2,* and *OSR2* genes in the SSB intermediate region, these are involved in tubule differentiation and expansion [8,47,48,52]. Hnf1b binds to the promoter regions of genes expressed in the proximal/intermediate tubule of SSB, including *CDH6*, *PCSK9,* and *TFAP2B* [48]. *CDH6* is similarly expressed in RV and proximal tubule precursor cells [8]. Hnf1b activates *HNF4A* transcription in the distal region of the CSB and the proximal region of the SSB [2,8,48,52]. *HNF4A* encodes a nuclear transcription factor required for proximal tubule development [48,52]. In the *Hnf1b*-altered model, Hnf4a is downregulated during all nephrogenesis stages and in mature kidneys [21]. This gene has been associated with diabetes and renal cyst development [36,38,63,64].
  In the Heliot C. et al. [48] study, the conditional inactivation of *Hnf1b* in murine nephron progenitors led to rudimentary nephrons comprising a glomerulus connected to the collecting system by a short tubule with distal fates. This defect was preceded by strong downregulation of the Notch pathway components (Lfng, Dll1, and Jag1) and Irx1/2 factors, which are potential regulators of proximal and loop of Henle segment fates (Figure 3c).

In summary, transcription factors such as Hnf1b do not regulate single genes but entire gene networks [34], and Hnf1b plays a central role in initiating nephrogenesis and driving the differentiation and expansion of the RV into a mature nephron [48,60].

### 4.2. Hnf1b Implication in Apical-Basolateral Polarity, Tight Junctions, Primary Cilia Development and Cyst Formation

Hnf1b-regulated components discussed previously are essential for the process of nephrogenesis, and some of them also play a role in maintaining polarity in different metanephron regions.

Nephrons are composed of renal epithelial cells organized in a monolayer, connected by adherent and tight junctions. Their proper functioning depends on maintaining apical–basolateral polarity [55], which ensures the correct positioning of cytoskeletal and junctional components [34]. Massa et al. [8] observed defects in the CSB and SSB stages in the absence of Hnf1b, highlighting its role in epithelial maturation and maintenance [8]. Moreover, altered polarization, differentiation, and organization of renal epithelial cells were noted in *Hnf1b*-inactivated models [34,60,65].

Par and Crumbs complexes are constituted by core proteins involved in establishing apical polarity, and are evolutionarily conserved [34,66]. The Par complex consists of Par3, Par6, Cdc42, and aPKC, while the Crumbs complex includes Crb3, Pals1, and Patj [66,67,68]. Basolateral polarity, on the other hand, is primarily regulated by the Scribble proteins: Scrib, Dlg, and Llgl [34,68].

Tholen et al. [34] identified Hnf1b-regulated genes involved in cell polarity, cell–cell junctions, and cytoskeletal integrity using a mouse DCT cell line. These genes include *PARD6B*, *CRB3*, *LLGL2*, *ANK3*, *ADD2*, *RAB3IP,* and *DSG2* [34]. Other Hnf1b-regulated genes contributing to apical–basal polarity include *ATP6V1B1*, *SLC26A4*, *PDZK1*, *AQP5,* and *HPN* [21,60]. They also reported reduced tight junction integrity and changes in markers of polarized epithelium such as Cdh16, Pkhd1, and Cys1 [60], alongside aberrant localization of Muc1, basal Laminin, and apical aPKC [34,48,60].

In the study by Niborski L et al. [21], reduced Hnf1b expression led to the downregulation of Spp1 and Spp2, proteins crucial for early PT stages, particularly in cell adhesion and the extracellular environment [1,5,21]. Another early PT marker downregulated under these conditions is *Cubn*, a gene linked to chronic benign proteinuria and Imerslund–Grasbeck syndrome [1,21,23,69].

Several studies suggest that defects in renal cell polarity can affect primary cilia maintenance, and ciliary dysfunction is closely associated with renal cyst formation and failure [2,3,34,60,70,71]. Indeed, renal cysts are frequently observed in *HNF1B* patients and animal models [14,19,72]. Otherwise, Hnf1b regulates genes implicated in primary cilium development [2]; they influence cell proliferation, migration, apoptosis, planar cell polarity, and differentiation via pathways such as Wnt, cAMP, and mTOR [3,34,72,73]. Key primary cilium genes regulated by Hnf1b include:

- *PKD2*: encodes Polycystin 2, a Ca^2+^-permeable cation channel [1,2]. *PKD2* mutations cause Autosomal Dominant Polycystic Kidney Disease (ADPKD) [3,23]. This is due to reduced Ca^2+^ entry and activation of the Ca^2+^-inhibitable adenylyl cyclases (AC5 and AC6), which elevates cAMP levels. This rise in cAMP stimulates cell proliferation and fluid secretion, promoting cyst growth [3,74].
- *PKHD1*: encodes a protein located in the primary cilia [2,3]; Hnf1b regulates this gene, particularly in collecting ducts (CD) [3]. *PKHD1* is associated with Autosomal Recessive Polycystic Kidney Disease (ARPKD) [3,23].
- *PDE4C*: catabolizes cAMP in the primary cilia and is downregulated in *HNF1B* mutant cells, leading to increased cAMP levels and subsequent cyst formation [3,74].
- *UMOD*: involved in electrolyte transport in the thick ascending limb (TAL) [2]; *UMOD* is linked to ADTKD and ciliary function [3].
- *KIF12*: part of the microtubule cytoskeleton [1,2,49], expressed in primary cilia and mitotic spindles [2,34]. It is downregulated in *HNF1B* patients [49], although no significant effect was observed in the murine model by Niborski L et al. [21].
- *HNF4A*: plays a role in various kidney processes, including PT development and cystogenesis, by modulating *PKD1* expression [63].
- *SOCS3*: negatively regulated by Hnf1b; its upregulation impairs tubule formation [3] and is highly upregulated in human polycystic kidneys [3,75].
- *BICC1*: an RNA-binding protein that modulates translation during embryonic development and is associated with renal cystic dysplasia [1,23,76].
- *CYS1*: involved in congenital polycystic kidney disease [1,21,69].
- *GLIS2*: encodes a transcription factor linked to nephronophthisis [3,23].
- *GLIS3*: implicated in pancreatic beta cells, thyroid, eye, liver, and kidney development [1]. It is also involved in polycystic disease affecting both the kidneys [21,69,77] and pancreas [78].

### 4.3. Hnf1b Regulates Ion Transport in Kidney

**Figure 4:**
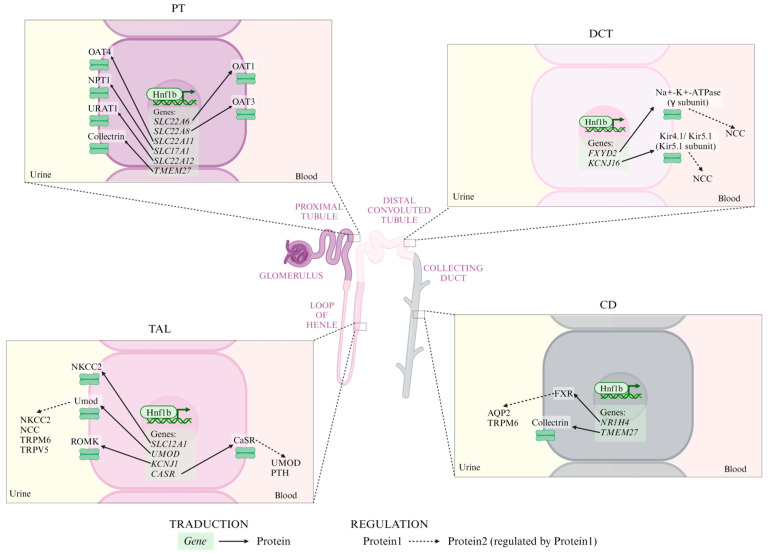
Ionic transporters regulated by Hnf1b in each tubular segment. Continuous arrows represent transduction processes from *gene* to protein; dashed arrows represent regulation processes from protein1 to protein2. PT: proximal tubule; TAL: thick ascending limb; DCT: distal convoluted tubule; CD: collecting duct; OAT4: organic anion transporter 4; NPT1: Na^+^-phosphate transporter 1; URAT1: urate transporter; OAT1: organic anion transporter 1; OAT3: organic anion transporter 3; NKCC2: Na^+^-K^+^-Cl^-^ co-transporter 2; Umod: uromodulin; ROMK: renal outer medullary potassium channel; NCC: Na^+^-Cl^-^ co-transporter; TRPM6: transient receptor potential melastatin type 6; TRPV5: TRP vanilloid type 5; CaSR: calcium-sensing receptor; PTH: parathyroid hormone; FXR: farnesoid X receptor; and AQP2: aquaporin 2.

In mature kidneys, Hnf1b regulates genes responsible for solute transport [23]. Alterations in *HNF1B* often lead to ADTKD-HNF1B, which is characterized by electrolyte imbalances such as hypomagnesemia, hyperparathyroidism, hyperuricemia, and hypocalciuria. These imbalances arise from Hnf1b’s transcriptional regulation of ion transport gene networks (Figure 4) [2,3,11,19,79].

In the proximal tubule (PT), Hnf1b regulates expression of:

- Organic anion transporters (*SLC22A6*, *SLC22A8,* and *SLC22A11* genes) [2,3,80,81,82].
- Na^+^-phosphate transporter 1 (*SLC17A1* gene) [2,3,83].
- Renal urate transporter (*SLC22A12* gene) [2,3,84].
- *TMEM27*, which encodes collectrin and enhances amino acid transporter surface expression. *TMEM27* is also expressed in the CD [85]. Its expression is downregulated in *Hnf1b*-altered mice [21]. *Tmem27* disruption in mice leads to a severe defect in renal amino acid uptake [3].

Most *HNF1B* patients do not show a strong PT phenotype. This may be due to Hnf1a compensation, which is expressed exclusively in the PT; other nephron segments do not benefit from this compensatory action [2,86].

In the TAL, Hnf1b regulates:

- *SLC12A1*, encoding the Na^+^-K^+^-Cl^-^ co-transporter 2 (NKCC2) [2,48], which is essential for Na^+^ and Cl^-^ transport across the apical membrane [3] and paracellular divalent cation transport [2]. Mutations in this gene are linked to Bartter syndrome [23].
- *UMOD*, which encodes uromodulin, activates NKCC2, Na^+^-Cl^-^ co-transporter (NCC) [87], transient receptor potential melastatin type 6 (TRPM6) [88] and TRP vanilloid type 5 (TRPV5) [89]. Reduced uromodulin expression is associated with medullary cyst formation and renal electrolyte imbalances [2,90].
- *KCNJ1,* which encodes a K^+^ channel and is associated with Bartter syndrome [1,21,23].
- *CASR* (calcium-sensing receptor, CaSR), a negative regulator of *UMOD* [2,91]. Decreased CaSR expression is expected to elevate blood calcium levels [2,3,11,92]. In the parathyroid gland, PTH expression can be repressed by Hnf1b or CaSR [2,93]. PTH also inhibits uric acid secretion via ABCG2 downregulation [2].

In the DCT, Hnf1b regulates:

- *FXYD2* encodes the γ subunit of the Na^+^-K^+^-ATPase. Mutations in *HNF1B* and *FXYD2* mainly result in hypomagnesemia, hypocalciuria, and basolateral membrane depolarization, leading to increased intracellular chloride levels and inhibiting NCC [2,3,14,24,79,94,95].
- *KCNJ16,* encoding the Kir5.1 subunit of the basolateral Kir4.1/Kir5.1 K^+^ channel, crucial for K^+^ recycling and Na^+^-K^+^-ATPase activity. Dysfunction of this channel leads to basolateral membrane depolarization and reduced NCC activity [2,96,97].

In the CD, Hnf1b regulates:

- *NR1H4,* encoding farnesoid X receptor (FXR), essential for urine concentration by regulating *AQP2* expression [2,98]. FXR also directly activates *TRPM6* expression, contributing to Mg homeostasis [2,99].
- *TMEM27,* which encodes an amino acid transporter (collectrin) which is also expressed in PT [85].

### 4.4. Role of Hnf1b in Intrarenal Metabolism: Mitochondrial Respiration and Cholesterol

A study by Casemayou et al. [49] identified *PPARGC1A* as a gene directly regulated by Hnf1b, as chromatin immunoprecipitation confirmed Hnf1b binding to *PPARGC1A* promoter, and inhibition or overexpression of Hnf1b led to decreased [45] or increased *PPARGC1A* expression, respectively [49]. *PPARGC1A* is a transcriptional coactivator that regulates mitochondrial biogenesis [1]. Its implication in the mitochondrial biogenesis mechanism provides an explanation for the observed metabolic change with *Hnf1b* inactivation or deletion in mouse PT cells [45,49], human cells [45], and kidney samples from patients with pathogenic *HNF1B* alterations [14]. The metabolic changes observed are similar to the Warburg effect seen under hypoxic conditions, and are characterized by increased lipid accumulation, oxidative stress modulators, lactate production, and decreased Ppargc1a and choline kinase-alpha expression, along with reduced ATP production [45,49]. 

This way, Hnf1b regulates the bioenergetic metabolism and mitochondrial morphology of renal tubular epithelial cells [2,3,45,49]. Given the kidney’s high energy demands to sustain basal metabolism and the electrochemical gradients necessary for active ion transport [2,100,101,102,103], compromised ATP production due to *HNF1B* pathogenic alterations can severely disrupt transporter function. This disruption can lead to energy metabolism alteration, fibrosis, acute kidney injury, electrolyte imbalances resembling a Gitelman-like phenotype, and progression to CKD [102,104].

Hnf1b also plays a role in renal cholesterol metabolism by directly regulating genes involved in cholesterol biosynthesis [2,3,105], such as *PCSK9,* a key gene which is crucial for nephrogenesis and internalization of low-density lipoprotein (LDL) receptors [45,106]. Hnf1b inactivation reduces cholesterol biosynthesis while increasing cholesterol uptake in the kidneys, potentially altering lipid metabolism [2,3,105].

## 5. Conclusions and Future Perspectives

Throughout this review, we have highlighted the diverse renal functions regulated by Hnf1b, including nephrogenesis, tubular elongation, epithelial cell polarization, tight junctions, primary cilia structure, ion transport, mitochondrial respiration, and cholesterol metabolism.

Genetic variants altering Hnf1b function lead to dysregulation of its target genes, resulting in a spectrum of renal and extrarenal pathologies. Figure 2 illustrates the Hnf1b-regulated genes with their associated diseases.

The variability in phenotype manifestation, progression, and severity is notable among individuals with *HNF1B* alterations. Studies have shown that both 17q12 deletions and point mutations in *HNF1B* cause MODY and renal anomalies (Figure 2). However, point mutations tend to result in a more severe and earlier renal phenotype, while 17q12 deletions are associated with a broader range of renal and non-renal disorders, including liver and psychiatric conditions [12,14,19,26,27,30,31].

Investigating the molecules that constitute the Hnf1b transcriptional complex in renal tissues is also essential. Even if *HNF1B* itself is unaltered, incomplete transcription complexes may still cause dysregulation and associated manifestations. Identifying these components and incorporating them into genetic screening could improve diagnostic accuracy and patient outcomes. 

In conclusion, *HNF1B* is a key regulator, especially in prenatal and postnatal kidneys, controlling genes involved in several physiological functions mentioned previously. Genetic *HNF1B* alterations can affect all of these processes, triggering different renal and extrarenal diseases. Further research into animal models and genotype–phenotype correlations in *HNF1B*-related disease is crucial. Understanding these correlations can help elucidate the full spectrum of the disease and guide more personalized treatment approaches. 

## Figures and Tables

**Table 1:** Index of mechanistic action of Hnf1b in renal physiology.

Subsection	Mechanistic Action
4.1.	Nephrogenesis. Identification of the genes regulated by Hnf1b at each stage of nephron formation and their contributions to kidney development.
4.2.	Apical-basolateral polarity, tight junctions, primary cilia development, and cyst formation. Role of Hnf1b in these processes.
4.3.	Ion transport. Hnf1b-regulated genes involved in tubular ion transport, a process crucial for maintaining electrolyte balance and renal function.
4.4.	Intrarenal metabolism. Hnf1b involvement in mitochondrial respiration and cholesterol metabolism.

## Data Availability

No new data were created or analyzed in this study. Data sharing is not applicable to this article.
